# Narcolepsy in Children and Adults: A Guide to Improved Recognition, Diagnosis and Management

**DOI:** 10.3390/medsci7120106

**Published:** 2019-11-27

**Authors:** Anne Marie Morse

**Affiliations:** Division of Pediatric Neurology, Janet Weis Children’s Hospital, Geisinger, Danville, PA 17820, USA; amorse@geisinger.edu; Tel.: +1-(570)-271-8524; Fax: +1-(570)-270-7923

**Keywords:** narcolepsy, cataplexy, orexin, hypocretin

## Abstract

Narcolepsy is a rare condition that affects children and adults, and commonly has an onset in childhood. Time to appropriate diagnosis frequently is at least a decade. Unrecognized or misdiagnosed symptoms of narcolepsy contribute to increased morbidity, disability and socioeconomic liability in these patients. Delays in diagnosis may be related to variability in presentation in childhood, lack of familiarity with symptoms or appropriate diagnostic testing or misdiagnosis with accidental introduction of treatment that may modify or mask narcolepsy features. Improved awareness about the diagnosis and tailored therapies improve clinical and socioeconomic outcomes by reducing time to effective treatment. Application of effective treatment results in long-term benefits by improving clinical outcomes, potentially enabling improved education, increased employment opportunity, and improved work productivity and quality of life. This review provides a comprehensive stepwise approach to improve knowledge and comfort for recognition of symptoms, diagnostic strategies and management considerations of narcolepsy in children and adults.

## 1. Introduction

Narcolepsy is a disabling neurodegenerative condition characterized by severe excessive daytime sleepiness (EDS). In fact, EDS is considered one of the most sensitive symptoms of narcolepsy, present in 100% of patients. There are two types of narcolepsy recognized: Narcolepsy Type 1 (NT1) and Narcolepsy Type 2 (NT2) [1]. Clinically, Narcolepsy Type 1 is characterized by the pentad features of EDS, sleep fragmentation, sleep-related hallucinations, sleep paralysis and cataplexy. Cataplexy are transient episodes of aberrant tone that are frequently provoked by strong emotion. Cataplexy is considered the most specific symptom for narcolepsy [2]. A patient may be defined as Narcolepsy Type 1, even if there is the absence of cataplexy, but there is evidence of hypocretin (orexin) deficiency in the cerebrospinal fluid (CSF). Narcolepsy Type 2 lacks the clinical features of cataplexy, and if CSF hypocretin is performed, it is normal. The perception of the symptoms of narcolepsy may seem wildly random, but are, in fact, synthesized by all representing REM (rapid eye movement sleep) dissociative features.

## 2. Inadequate and Delayed Diagnosis

It is estimated that about 1 in 2000 individuals are affected, but up to 50% of individuals may currently be undiagnosed [3,4,5]. Diagnosis frequently can take as long as 8–10 years to be achieved. Cataplexy presence as an initial symptom, however, is generally associated with a shorter time to diagnosis [6]. Onset of symptoms typically occurs in a bimodal distribution with an initial main peak at 15 years old and a lesser second peak at approximately 35 years old [7,8].

The burden of narcolepsy can vary with the age of onset and time to diagnosis. Children and adolescents frequently suffer from poor school performance, strained family relationships, depressed feelings, impaired social life and even isolation [6]. Adults frequently achieve a lower earning potential and employment rates, increased psychiatric and medical co-morbidity, higher degrees of disability [9].

An investigation of 1000 patients with narcolepsy showed a median onset of symptoms at 16 years, but the median age of diagnosis of 33 years [10], illustrating the significant delay in diagnosis. 

Misdiagnosis and comorbidity can contribute to the delay in diagnosis of narcolepsy. Psychiatric comorbidity can present a unique challenge in that both the symptoms and the treatment of psychiatric disease can lead to masking of narcolepsy symptoms [11]. Review of the Nexus Narcolepsy Registry revealed that nearly 60% of participants received at least one misdiagnosis before having narcolepsy identified [12]. 

## 3. Approach to Symptom Recognition

Lack of awareness and ability to recognize the cardinal symptoms of narcolepsy contributes to inability to appropriately diagnosis the condition. The AWAKEN survey in 2014 clearly highlights the frequent inability for physicians to recognize these symptoms. Unfortunately, this study found that less than 10% of primary care providers and only 22% of sleep specialists could identify all five narcolepsy symptoms [13]. Improved knowledge of these symptoms, including recognition of the clinical manifestations of these symptoms, may lead to improved time to diagnosis and optimized treatments. 

Providers should consider use of validated measures to help identify patients with features of narcolepsy, especially if there is reduced confidence in the ability to recognize or identify features. The Epworth sleepiness scale (ESS) and the ESS-child and adolescent version (ESS-CHAD) are validated measures in adults and children to identify sleepiness [14,15]. Patients who score greater than 10 are considered sleepy, however, those scoring greater than 16 are generally considered high-risk for hypersomnia disorders. Anic-Laba et al. validated the cataplexy questionnaire to guide easy identification of those who are high or low risk for cataplexy [16]. The Swiss Narcolepsy Scale is a validated 5-item patient reported scale used identify patients who are high likelihood for narcolepsy and is available for use for free at https://www.swissnarcolepsyscale.com/ [17]. It is important to compliment the use of these validated scales, with subjective input from the patient regarding the impact of symptoms on quality of life and disability. This information generally proves to be more valuable in targeting goals that are meaningful to the patient for improvement and adherence to treatment. 

### 3.1. Pediatrics

**Figure 1:**
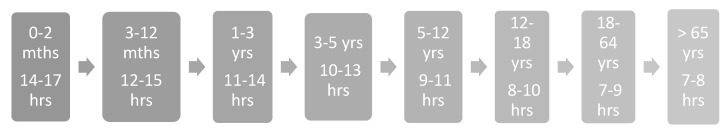
Age-appropriate sleep hours.

Although excessive daytime sleepiness is generally the primary presenting symptom recognition of these symptoms may be challenging in children and adolescents due to varying clinical features. EDS may present as greater than age-appropriate sleep hours (Figure 1) [18], reintroduction of daytime napping or irrepressible sleep attacks in sedentary settings. Other, sometimes less obvious, manifestations may include hyperactivity, mood disturbance, aggressiveness, and irritability either as compensation or a fight against sleepiness. Due to the various manifestations of EDS, medical and psychiatric care may be sought for alternative concerns, including deteriorating school performance, worry of substance use disorder, family strain, and reduced academic or social participation. When sleepiness is present, identify timing of onset, especially in the setting of an explosive onset of symptoms. NT1 frequently has a recognized onset in spring or summer, and may have a preceding history of upper respiratory infection, influenza or streptococcal infection [19,20].

In general, providers may be challenged to accurately identify features of cataplexy. This challenge can be compounded in children and adolescents where the spectrum of clinical features is widened and extends beyond the typical episodes of emotionally provoked episodes of transient hypotonia. Postiglione et al. provides a detailed description, including photographs, of the additional cataplexy features that can be seen in pediatric narcolepsy including “cataplectic facies”, hyperkinesis or active motor phenomena and even complex movement disorder (Table 1) [6,16,21].

Other features of narcolepsy in childhood may be overlooked, misdiagnosed or considered typical for childhood. For instance, sleep-related hallucinations or REM behavior disorder may be perceived as nightmare disorder or other non-REM parasomnia [22]. Sleep fragmentation, nocturnal awakenings, and poor sleep efficiency may be considered behavioral, hormonal or related to sleep disordered breathing, especially when there is significant weight gain accompanying the onset of symptoms. It is important to highlight that this challenge is compounded by the fact that children with narcolepsy have a 1000 times higher prevalence of precocious puberty and double the prevalence of obesity compared to the general population [23].

### 3.2. Adults

Identifying symptoms of narcolepsy in adults can be difficult due to comorbidity and misdiagnosis, medications used for other indications that may mitigate narcolepsy symptoms, and underreporting of features due to acquired normalization of symptoms experienced. EDS in adults frequently presents with complaints of irrepressible need to nap when sedentary, mental slowing or clouding, and even injury, such as motor vehicle accidents related to sleep attacks. However, patients with narcolepsy may rationalize these symptoms based on other diagnoses they have received or underestimate the severity due to compensatory actions. For instance, lack of motivation and wakefulness due to sleepiness or scheduling days to accomplish more during recognized wakeful periods of the day. When sleepiness is present it is critical to identify timing of onset, which may be even a decade preceding, to identify preceding illness or head trauma that may increase risk for narcolepsy. 

Adults with narcolepsy who experience cataplexy, may experience either or both partial or generalized cataplexy. Cataplexy in adults is typically described as emotionally provoked transient episodes of loss of tone, but can also occur independent of emotion. In adults who experience cataplexy, recognition of these symptoms may be poor. In fact, many may refer to themselves as clumsy or have unwittingly developed avoidance techniques to mute emotional experience. It is critical to re-evaluate for symptoms of cataplexy at every visit for individuals who have excessive daytime sleepiness, even those who may have been diagnosed with another sleep disorder, such as sleep apnea, to ensure that the diagnosis of narcolepsy is not missed. 

Evaluation for other supporting evidence of narcolepsy and REM dissociative behavior including sleep paralysis, sleep-related hallucinations, automatic behavior, sleep onset vivid dreaming and REM behavior disorder (RBD) should be performed. Automatic behavior is daytime lapses of awareness or a dream-like state of being engaged in activity that are poorly recalled by the patient [24]. An example would be a mother who is cooking dinner, who has a dream-like state of cooking, but lack of awareness of activity and reaches into oven to take out dinner without oven mitts and becomes alert with the burning heat. It is not uncommon for this to be misdiagnosed or misinterpreted as a focal seizure. Sleep-onset vivid dreaming is eliciting the clinical history that if sleeping for 15 min or less one would have a vivid dream. This is a clinical indicator of sleep-onset REM sleep.

## 4. Approach to Diagnosis

### 4.1. Sleep Testing

The primary sleep testing utilized to evaluate hypersomnia disorders is nighttime in-lab polysomnography (PSG) and the following day multiple sleep latency test (MSLT). In-lab polysomnography must be performed prior to the MSLT and it is recommended that at least 6 h of sleep are captured prior to performing MSLT for optimal sensitivity and specificity [25]. The polysomnography may provide additional diagnostic clues for a diagnosis of narcolepsy. 

The MSLT is four to five opportunities to nap every 2 h, evaluating time to fall asleep or sleep latency (SL) and whether the patient experiences sleep-onset REM periods (SOREMPs). SOREMPs are the presence of REM sleep within 15 min of sleep onset, as opposed to the typical cycle taking about 90–120 min [26]. Narcolepsy Type 1 and Type 2 are generally diagnosed based on the presence of EDS and findings of an average SL of ≤ 8 min and the presence of two or more SOREMPs on sleep testing [27]. It is important to consider factors that may influence the yield of the MSLT (Box 1). In children, it is important to recognize that normal sleep latency may vary by tanner staging [28] and may contribute to diagnostic difficulty. Sleep diaries and/or actigraphy are recommended for at least 2 weeks prior to PSG/MSLT, to identify features of insufficient sleep or delayed-sleep-phase syndrome that can produced reduced SL or SOREMPs leading to false-positive testing for narcolepsy [25].

Box 1 **Multiple sleep latency test (MSLT) checklist to optimize results and interpretation.**

✓ Prepare patient for length of study and description of the overnight PSG and the MSLT.
✓ Evaluate medications to determine if any influence sleep latency or are REM suppressants.
✓ Is it safe to temporarily discontinue these medications?
  ○ If so, wean medication with plans to be off medication for at least 5 half-lives or 2 weeks if half-life unknown.
✓ Complete sleep diaries and/or actigraphy to document usual sleep wake patterns
  ○ Consider optimizing sleep schedule first if shift work, delayed sleep phase or chronic insufficient sleep is present.
✓ Ensure PSG demonstrates at least 6 h of total sleep time prior to MSLT.
✓ Evaluate PSG for additional supportive evidence of narcolepsy.
  ○ Sleep fragmentation, RBD, REMWA, increased PLMs, SOREMP.
✓ Consider Tanner Staging and age of patient when evaluating average sleep latency.
  ○ If study is borderline and history is convincing, consider repeat study in 6 months versus CSF hypocretin (orexin), if suspicious on NT1.
✓ Attempt to keep patient awake between naps during MSLT.
  ○ If unable to do so, consider continuous recording during MSLT to evaluate sleep wake pattern.*

NT1—narcolepsy type 1; PLMs—periodic limb movements; RBD—REM behavior disorder, REMWA—REM without atonia; REM—rapid eye movement sleep; CSF—cerebrospinal fluid. * Note: there is no standard for scoring this method.

### 4.2. Human Leukocyte Antigen (HLA) Testing

There are specific HLA haplotypes that have been identified as either protective or as a risk factor for narcolepsy [29,30,31,32]. HLA-DQB1*0602 has been found in 85–95% of patients with narcolepsy. [31] The risk to develop Narcolepsy Type 1 is about 7-fold higher in those who are heterozygous and up to 25-fold higher in those who are homozygous for HLA-DQB1*0602 [30]. In families with a member with narcolepsy, the estimated risk for family members to develop narcolepsy is suggested to be 10–40 times that of the general population [33]. 

Although genomic burden influences risk, it has not been shown to influence course or disease burden [30]. Only about half of individuals with Narcolepsy Type 2 may be HLA-DQB1*0602-positive [32]. In addition, this haplotype has also been identified in 12–38% of the general population without narcolepsy [31]. The lack of specificity has led to HLA testing not routinely being advocated to identify the diagnosis of narcolepsy. However, the presence or absence of a specific haplotype may provide excellent diagnostic clues, especially in cases with multiple co-morbidities or medications contributing to an ambiguous presentation.

### 4.3. Cerebrospinal fluid (CSF) Testing

Cerebrospinal fluid (CSF) evaluation may identify low orexin/hypocretin, less than 110 pg/mL, in 87–96% of patients with Narcolepsy Type 1 [31,34]. Narcolepsy Type 2 has normal CSF orexin/hypocretin. The lack of cataplexy and normal CSF orexin/hypocretin in Narcolepsy Type 2 can contribute to difficulty in recognition and distinguishing from idiopathic hypersomnia. CSF evaluation is a new clinically available tool that many providers are uncertain when to consider use (Box 2. It is not uncommon for sleep providers to partner with either interventional radiology, neurology, anesthesia or hematology oncology to perform the lumbar puncture services.

Box 2 **Clinical scenarios to consider CSF orexin testing.**

Non-diagnostic PSG/MSLT testing in a patient with cataplexy and EDS
Non-diagnostic PSG/MSLT testing in a patient without cataplexy, EDS and HLA+
Non-diagnostic PSG/MSLT testing in a patient with EDS, HLA+, +/− cataplexy, who is unable to discontinue REM suppressing/sleep influencing medications due to safety/medical concerns
Pediatric patients at extreme ages (i.e less than 5 years old)
Pediatric patients with abnormal SL based on tanner stage, but non-diagnostic based on criteria

## 5. Approach to Treatment

Treatment for patients with narcolepsy should be individualized and tailored to patient-identified disability and goals of care. Re-evaluation of symptom presence and control at each visit, as well as surveillance for side effects, are critical for optimal adherence and improvement. When discussing pharmaceutical therapy, it is necessary to include information on convenience of administration, efficacy, risk for abuse, adverse effects and impact on comorbidities. It is important to complement pharmaceutical (Table 2) with behavioral interventions (Table 3) to maximize symptom improvement. 

### 5.1. Pharmacologic Strategies

Pharmaceutical therapy is generally directed at treating excessive daytime sleepiness, cataplexy and sleep fragmentation. Excessive daytime sleepiness is generally managed with either wake-promoting agents (e.g., modafinil and armodafinil, solriamfetol, pitolisant), traditional stimulants (methylphenidate and amphetamines) or sodium oxybate. Wake-promoting agents and sodium oxybate are considered standard-of-care therapy for the treatment of excessive daytime sleepiness in narcolepsy [44]. Sodium oxybate is FDA-approved in adults and pediatrics (ages 7 and older) and is the only treatment for EDS that also benefits symptoms of cataplexy [45]. Traditional stimulants are considered second- and third-line therapies for the treatment of EDS [44]. Solriamfetol and pitolisant are newer agents, but are likely to become first-line considerations for EDS [46,47].

Anti-cataplectic agents are typically agents that target norepinephrine modulation and include tricyclic anti-depressants, selective serotonin norepinephrine reuptake inhibitors (SNRI), selective serotonin reuptake inhibitors (SSRI), atomoxetine and sodium oxybate. Sodium oxybate is the only agent with an FDA approval for cataplexy treatment. [45] With that said, SNRIs and SSRIs are probably the most frequently prescribed antic-cataplectics, with venlafaxine being favored. It is important to note that an abrupt withdrawal of SSRI or SNRI can lead to rebound cataplexy, which is not seen with sodium oxybate [46]. 

### 5.2. Non-Pharmacologic Strategies

Non-pharmacologic strategies (Table 3) may have specific symptoms that they target (e.g., mitigate sleep inertia), but frequently are oriented to optimizing overall functioning, depression, anxiety, and improved quality of life. There is less robust data to support or negate the efficacy of use of non-pharmacologic strategies and the data available may or may not be specific to narcolepsy. It is important to consider these non-pharmacologic strategies, especially in the school or workplace. School-aged children are eligible for either a 504 plan or individualized education plan based on the diagnosis of narcolepsy, which would allow implementation of behavioral strategies to optimize school success [48]. Adults are protected under the Americans with Disabilities Act and can, therefore, request reasonable accommodations at work [48,49].

## 6. Conclusions

Narcolepsy is a devastating neurologic process that carries a high degree of disability, comorbidity and cost, which is augmented by poor physician recognition and delayed diagnosis [9,50]. Recognition of varying features of excessive daytime sleepiness across age groups is critical, as EDS is present in 100% of all narcolepsy patients. Careful questioning to solicit evidence of cataplexy can be key to uncovering many patients with narcolepsy. However, recognizing that cataplexy is not always present, one should adopt an organized and thorough approach to identify other REM dissociative features suggestive of narcolepsy.

Diagnostic strategies must be tailored based on the limitations present in the individual being evaluated. Opportunity to utilize CSF testing in individuals who cannot complete MSLT testing or have borderline test results increase the opportunity to capture patients who may otherwise be missed. However, caution should be made to not exclude consideration of NT2 or idiopathic hypersomnia with CSF testing is normal.

Management should always be a combination of behavioral and pharmacologic strategies that are tailored to the patient and their current life experience and expectations. Careful assessment of impact of symptoms on academics/work, socialization, safety, intimacy and relationships can help guide appropriate recommendations. Regular follow up is important to ensure adherence and continued safety, but also to monitor for evolving symptoms.

## Figures and Tables

**Table 1:** Features of cataplexy observed in adults and pediatrics [6,16,21].

Type	Description	Example
Negative	Transient loss of antigravity muscle tone, frequently evoked by emotion. Near continuous hypotonia without emotional stimulus	Generalized collapse to the ground with preserved awareness, knee buckling, loss of tone in hands, head drop. General floppiness, abnormal/semi-ataxic gait.
Active	Hyperkinetic features that may be enhanced by emotional stimuli. Complex Movement disorder.	Perioral/tongue movements, facial grimacing, eyebrow raising. Tic like stereotyped motor movements
Mixed	“Cataplectic Facies”	Facial hypotonia with ptosis, mouth opening and tongue protrusion.

**Table 2:** Pharmaceutical therapy for narcolepsy.

Symptoms Treated	Drug	FDA Approval (Ages)
Excessive Daytime Sleepiness	Modafinil	Yes (18 years and older)
	Armodafinil	Yes (18 years and older)
	Sodium Oxybate	Yes (ages 7 years and older)
	Methylphenidate	Yes (ages 6 years and older)
	Dextroamphetamine	Yes (ages 6 years and older)
	Solriamfetol	Yes (18 years and older)
	Pitolisant	Yes (18 years and older)
Cataplexy	Sodium Oxybate	Yes (ages 7 years and older)
	Venlafaxine	No
	TCA * (e.g., protryptiline, clormipramine)	No
	SSRI* (e.g fluoxetine)	No
	Atomoxetine **	No
EDS + Cataplexy	Sodium Oxybate	Yes (ages 7 years and older)

* SSRI—selective serotonin reuptake inhibitor; TCA—tricyclic antidepressant. ** slight wake-promoting benefit.

**Table 3:** Non-pharmacologic strategies for narcolepsy.

Behavioral Strategy	Description
Strategic Caffeine	Plan use of caffeine intake to promote performance and alertness [35]
Sleep Hygiene	Sleep related behaviors to enhance and achieve age appropriate number of hours of sleep [36]
Sleep Scheduling	Regular sleep–wake schedule [36]
Cognitive Behavioral Therapy	Systematic application of techniques needed to evaluate and improve behavior [37]
Scheduled napping	Nap that is scheduled during individuals typical height of sleep inertia [38]
Strategic napping	Planned nap of specific duration to promote performance and alertness [39]
Support Groups	In person or online social communities for support [40]
Exercise	Any cardiovascular activity for physical engagement [41]
Mindfulness	Meditation and self-awareness [41]
Yoga	breath control, simple meditation, adoption of specific postures for health/relaxation [41]
Diet	Small, frequent meals to mitigate post-prandial. Low carbohydrate, ketogenic diet [42]
Temperature Manipulations	Cold temperature environments and avoidance of hot environments [43]
